# Novel Prion Protein in BSE-affected Cattle, Switzerland

**DOI:** 10.3201/eid1805.111824

**Published:** 2012-05

**Authors:** Reinhold Kittelberger

**Affiliations:** Biosecurity New Zealand, Upper Hutt, New Zealand

**Keywords:** bovine spongiform encephalopathy, BSE, prion protein, scrapie, transmissible spongiform encephalopathy, truncated fragment, Western blot, Prions and related diseases

**To the Editor:** In a recent issue of Emerging Infectious Diseases, Seuberlich et al. (*1*) reported a novel prion protein in cattle with bovine spongiform encephalopathy (BSE). Two cows in Switzerland, 8 and 15 years of age, tested positive in 2 approved screening tests, the PrioSTRIP test and the Prionics Check WESTERN (Prionics, Zurich, Switzerland). According to World Organisation for Animal Health guidelines, the 2 cattle are considered BSE positive. Histopathologic and immunohistochemical results were inconclusive because the tissues were severely autolyzed. Clinical signs were absent or the clinical history was not known.

After further analysis of brain tissues by using several monoclonal antibodies in a Western blot (WB), the authors concluded that they had identified an N-terminal truncated protease-resistant prion protein (PrP^res^) fragment that differs from the PrP^res^ fragments in 3 known types of BSE. No reference was made to the existence of N-truncated fragments, such as C1, of the normal prion protein PrP^C^, which have been reported for humans (*2**,**3*), mice (*4*), and cattle and other ruminants (*5*). The pattern in the WB of the novel prion protein (*1*) appears similar to that of the fragment C1 of the normal prion protein (*2**–**5*). The C1 fragment is more protease resistant than the intact PrP^C^ fragment because the protein part is more protected by the polysaccharide residues. Could it be that in the case of the severely autolyzed tissues of the cows in Switzerland, the proteinase K might already have been weakened or inhibited and when combined with the higher protease resistance of the C1 fragment, the digestion was incomplete?

Ten years ago, I looked at nonspecific, unusual samples from fallen stock cattle in New Zealand. Samples from these cattle had been confirmed as negative by paraffin-embedded tissue blot (University of Göttingen, Göttingen, Germany), sodium phosphotungstic acid precipitation, followed by WB (European Union Reference Laboratory for Transmissible Spongiform Encephalopathies, Veterinary Laboratories Agency, New Haw, UK), Prionics WB (Prionics AG, Zurich, Switzerland), histopathologic examination, and immunohistochemical testing (Veterinary Laboratories Agency). We became aware of such samples when the proteinase K digestion did not work properly (Figure). Unusual samples 1 and 2 contained increased amounts of a truncated fragment of normal PrP^C^, which was digested completely after the proteinase K concentration was increased.

**Figure:**
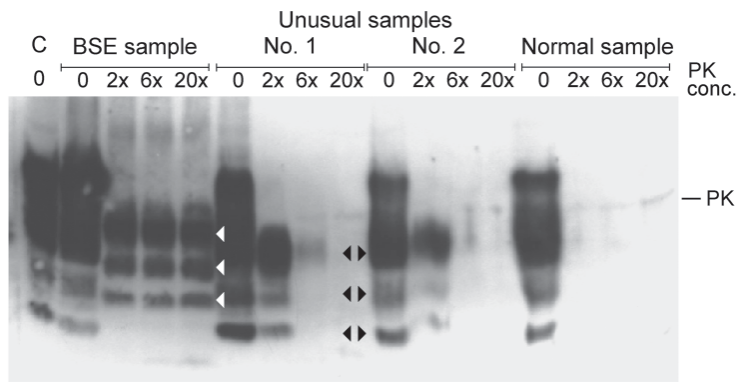
Western blot analysis of proteinase K (PK) digested brain stem samples with increasing concentrations of PK (relative to concentration used in the Prionics Check WESTERN (Prionics, Zurich, Switzerland). C, kit control, normal bovine brain homogenate; BSE, bovine spongiform encephalopathy sample from cow from Switzerland; unusual samples 1 and 2 and normal sample are from New Zealand cattle and had been confirmed as negative by several test methods (see text). Unusual samples 1 and 2 show a higher concentration of a truncated fragment of the normal prion protein, which was completely digested at increased PK concentrations. It is typical for this fragment that bands are identical in size to lower bands of the normal prion protein in the undigested samples of normal brain homogenates (black arrowheads). White arrowheads show major bands of PK-digested BSE prion protein.

I am convinced that the novel PrP^res^ described in article by Seuberlich et al. (*1*) is indeed a truncated fragment of the normal bovine PrP^C^ protein. Therefore, I would like to ask the editor to address the following issues with the authors: Why were no references to truncated fragments of PrP^C^ made in their article? Why was no WB analysis performed in which the novel PrP^res^ was shown next to normal, undigested PrP^C^ for band-size comparison? Why were no WB analyses shown in which the proteinase K concentration was increased?

It is laudable that in vivo transmission studies using transgenic mouse models and cattle are under way, which will sort out these findings conclusively. I expect that no disease development will be shown. Meanwhile, announcing new types of BSE is purely speculation.
