# Supplementary material for: Novel Prion Protein in BSE-affected Cattle, Switzerland
Source: Emerg Infect Dis. 2012 May;18(5):890–2. doi: 10.3201/eid1805.111824 (PMC3358048; doi:10.3201/eid1805.111824)
Supplement: Related texts

# Reply: Novel prion protein in BSE-affected cattle, Switzerland.

**DOI:** 10.3201/eid1805.120226

**Authors:** Torsten Seuberlich

**Affiliations:** University of Berne, Berne, Switzerland

**In Response:** Dr Kittelberger comments on our recent report of 2 cows in Switzerland that were classified as positive for bovine spongiform encephalopathy (BSE), according to the established criteria (*1*,*2*). He raises concerns that the unusual prion protein signature in Western blot (WB) in these cows represents a physiologic prion protein (PrP^C^) fragment, inefficiently degraded by proteinase K (PK), termed C1. Certainly the effects of tissue autolysis on PK activity and the molecular prion protein signature are of particular concern and deserve full consideration in data interpretation. In our study, molecular mass comparisons between PrP^C^ in non-PK–treated brain tissue of healthy cattle and the prion protein in samples from the 2 aberrant cows with BSE in WB were considerably hindered by overlapping C1- and full-length PrP^C^ bands in the non-PK–treated samples and did not allow for a robust conclusion (T. Seuberlich, unpub. data). It is noteworthy that the Prionics Check WESTERN (Prionics, Zurich, Switzerland) test has been extensively validated in terms of the diagnostic specificity, also on severely autolytic specimens (*3*–*5*). In none of these studies was a similar prion protein signature observed. We therefore considered it unlikely that the findings in the cases from Switzerland resulted from tissue autolysis.

Dr Kittelberger provides data from New Zealand cattle that revealed a similar prion protein signature in WB. He assumes that these animals had a negative BSE status and that the PK digestion in the WB did not work properly, which is supported by results from other diagnostic techniques. However, information about the degree of autolysis of these samples is missing, and, most notably, whether these findings are correlated with prion infectivity is not known. Strikingly, in contrast to the results for the samples from Switzerland, the samples from New Zealand are reported to be negative in the Prionics Check WESTERN. It would be fascinating to perform a side-by-side analysis of the samples from Switzerland and from New Zealand to determine whether the banding characteristics in both groups are identical. Studies are under way in our laboratory to further investigate the effect of tissue autolysis on PK activity and PrP^C^ degradation under experimental conditions. If our findings turn out to be the result of inhibited PK activity in BSE-negative cattle samples, the current diagnostic criteria might require revision. As long as the results of these experiments and the ongoing transmission studies are not available, we can neither confirm nor reject a novel type of BSE.

## References

[1] Seuberlich T, Gsponer M, Drögemüller C, Polak MP, McCutcheon S, Heim D, Novel prion protein in BSE-affected cattle, Switzerland. Emerg Infect Dis. 2012;18:158–9. 10.3201/eid1801.11122522261120PMC3310124

[2] Kittelberger R. Novel prion protein in BSE-affected cattle, Switzerland [letter]. Emerg Infect Dis. 2012;18:890–2 .10.3201/eid1805.11182422515935PMC3358048

[3] Schaller O, Fatzer R, Stack M, Clark J, Cooley W, Biffiger K, Validation of a western immunoblotting procedure for bovine PrP(Sc) detection and its use as a rapid surveillance method for the diagnosis of bovine spongiform encephalopathy (BSE). Acta Neuropathol. 1999;98:437–43. 10.1007/s00401005110610541864

[4] European Food Safety Authority. Scientific report on the evaluation of seven new rapid post mortem BSE tests. EFSA Scientific Report. 2004;18:1–13 [cited 2012 Mar 1]. http://www.efsa.europa.eu/en/scdocs/doc/18r.pdf

[5] Office International des Epizooties. OIE procedure for validation and certification of diagnostic assays. Abstract sheet for the Prionics-AG Check WESTERN [cited 2012 Mar 1]. http://www.oie.int/fileadmin/Home/eng/Our_scientific_expertise/docs/pdf/Abstract_20sheet_OIE_20Register_PrionicsWB_v1.pdf
